# Voxel-based spatial distribution of intracranial meningioma subtypes and their relationship to radiogenomic maps

**DOI:** 10.1093/braincomms/fcag025

**Published:** 2026-01-30

**Authors:** Georgios Naros, Aldo Spolaore, Sophie Wang, Mykola Gorbachuk, Kathrin Machetanz, Benjamin Bender, Felix Behling, Jens Schittenhelm, Marcos Tatagiba

**Affiliations:** Department of Neurosurgery and Neurotechnology, Eberhard Karls University, 72076 Tuebingen, Germany; Department of Neurosurgery and Neurotechnology, Eberhard Karls University, 72076 Tuebingen, Germany; Department of Neurosurgery and Neurotechnology, Eberhard Karls University, 72076 Tuebingen, Germany; Department of Neurosurgery and Neurotechnology, Eberhard Karls University, 72076 Tuebingen, Germany; Department of Neurosurgery and Neurotechnology, Eberhard Karls University, 72076 Tuebingen, Germany; Department of Neuroradiology, Eberhard Karls University, 72076 Tuebingen, Germany; Department of Neurosurgery and Neurotechnology, Eberhard Karls University, 72076 Tuebingen, Germany; Hertie Institute for Clinical Brain Research, Eberhard Karls University, 72076 Tuebingen, Germany; Center for CNS Tumors, Comprehensive Cancer Center Tuebingen—Stuttgart, 72076 Tuebingen, Germany; Center for CNS Tumors, Comprehensive Cancer Center Tuebingen—Stuttgart, 72076 Tuebingen, Germany; Department of Neuropathology, Eberhard Karls University, 72076 Tuebingen, Germany; Department of Neurosurgery and Neurotechnology, Eberhard Karls University, 72076 Tuebingen, Germany

**Keywords:** meningioma, voxel-based lesion symptom mapping, histological subtype, radiogenomics, spatial distribution

## Abstract

Meningiomas are histologically and genetically heterogeneous tumors with varying anatomical distributions. While distinct genetic mutations have been associated with specific tumor locations, the spatial distribution of histological subtypes and their relationship to radiogenomic profiles remains poorly defined. Moreover, the predictive value of spatial information for histopathological classification and tumor grading has not yet been systematically explored. This study aimed to systematically analyze the anatomical predilection of histological meningioma subtypes and their concordance with known mutation-specific spatial patterns, and the predictive potential of voxel-based spatial features. We retrospectively analyzed 737 patients undergoing surgical resection of intracranial meningiomas. Preoperative magnetic resonance images were normalized to a common stereotactic space, and tumors were semi-automatically segmented. Voxel-based lesion-symptom mapping (VLSM) was performed to identify subtype-specific spatial clustering. Spatial distributions were compared with mutation maps from current literature using receiver operating characteristic analysis (AUC-ROC). Additionally, multinomial logistic regression models were applied to evaluate whether tumor localization could predict histological subtype and World Health Organization (WHO) grading. Histological subtypes showed distinct spatial preferences. Meningothelial meningiomas clustered in the anterior and middle skull base; fibrous and transitional types predominated in the convexity, falx, and tentorium; secretory tumors localized to the sphenoid wing and petroclival region; and atypical meningiomas were common in the anterior falx and frontal convexity. Psammomatous meningiomas displayed a broader distribution with involvement of the petrous bone and foramen magnum. AUC analysis revealed strong concordance between histological subtypes and mutation maps, confirming known histogenomic associations (e.g. *KLF4/TRAF7* with secretory; *NF2* with fibrous and transitional; *SMO* with meningothelial). No associations to any mutation map were observed for angiomatous, microcystic and metaplastic meningiomas. Predictive modeling based solely on spatial features achieved moderate accuracy for subtype classification and higher accuracy for WHO grade prediction. Meningioma subtypes show distinct, statistically robust anatomical predilections that align with known genetic mutation maps. Predictive modeling highlights that spatial features themselves hold diagnostic and prognostic value, linking anatomical localization to tumor biology and aggressiveness. The study introduces anatomically precise voxel-based templates that may improve radiogenomic classification and non-invasive genotype prediction.

## Teaser

Naros *et al*. report that voxel-based spatial mapping of 737 intracranial meningiomas reveals distinct anatomical clustering of histological subtypes and CNS grades. The study shows that higher-grade tumors preferentially occur at the frontoparietal transition zone, linking tumor location with underlying genetic drivers and enabling anatomically precise radiogenomic classification.

## Graphical Abstract

**Graphical Abstract:**
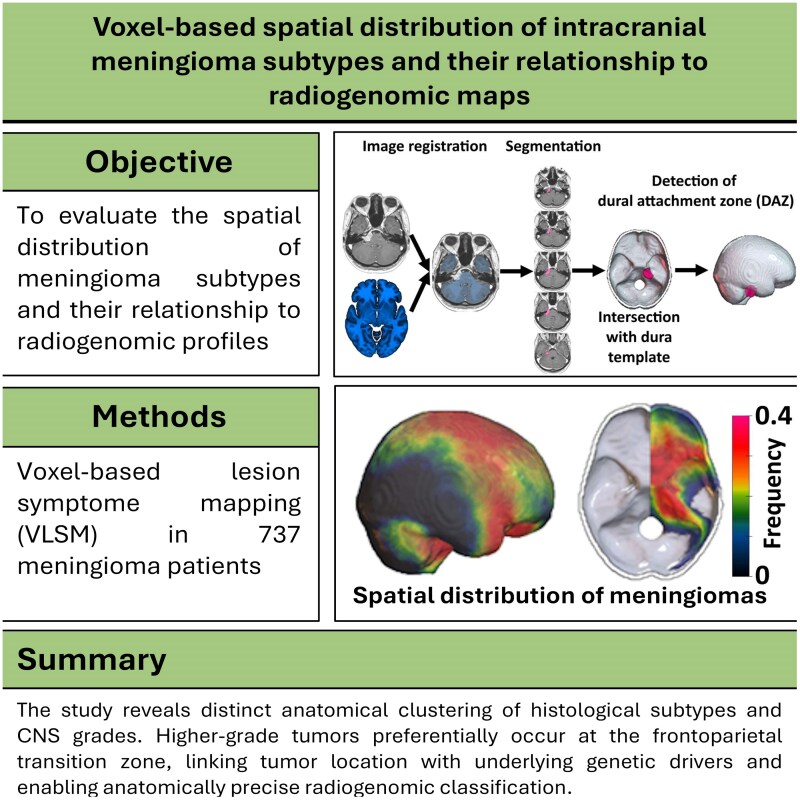

## Introduction

Meningiomas are the most common primary intracranial tumor in adults,^1^ originating from arachnoidal cap cells of the leptomeninges.^2^ In recent decades, several genetic mutations were identified that are associated with both the tumorigenesis of meningiomas^3^ and the oncological outcome after treatment.^4,5^ These factors contribute to the development of different histological phenotypes and the frequently updated World Health Organization (WHO) gradings.^2,3,6^ Meningiomas linked to deletions on chromosome 22q (affecting *NF2* or *SMARCB1*) are commonly associated with fibrous, psammomatous, transitional, atypical and anaplastic histological subtypes.^7,8^  *NF2-*mutated meningiomas represent the majority of tumors located in the falx cerebri, tentorium cerebelli, as well as cerebral and cerebellar convexities.^8-11^ Mutations in the PI3K-AKT-mTOR pathway (including *AKT1, AKT3, PIK3CA, PIK3R1*) have been observed in CNS WHO grade I meningothelial or transitional meningiomas arising from the medial skull base.^12,13^ Additionally, mutations affecting the Hedgehog signaling pathway (such as *SMO, SUFU, PRKA-R1A*) are more likely to result in CNS WHO Grade 1 meningothelial subtype located in the midline of the anterior cranial fossa.^8-10^ Furthermore, there are several other pathogenic variants (including *KLF4, TRAF7, SMARCE1, BAP1*) associated with secretory, meningothelial, clear cell or rhabdoid histology subtype of skull base meningiomas.^9^

Despite these findings, data on the spatial distribution of meningioma subtypes and their relationship to genetic factors and embryological origins remain limited and inconclusive.^9^ The location of intracranial meningiomas is generally described by their dural attachment zone (DAZ), but in large tumors (such as those in the falx or sphenoid wing), this can be obscured, and in some cases (such as convexity meningiomas), the DAZ description can be imprecise. Voxel-based lesion-symptom mapping (VLSM) offers a promising tool to investigate these complex relationships. This imaging-based statistical technique enables the spatial localization of disease patterns by normalizing individual tumor masks into a standard stereotactic space. VLSM allows for objective, rater-independent analyses of tumor location across cohorts, facilitating the identification of anatomical clusters associated with particular histological and clinical variables. Although VLSM has been widely applied in focusing on stroke and other focal brain lesion, its application in neuro-oncology—and specifically in meningioma research—remains limited. Only a few prior studies have employed this approach to characterize the spatial distribution of intracranial meningiomas,^14,15^ and even fewer have integrated histopathological or genetic data into the analysis.^16^

The present study seeks to address these gaps by applying voxel-based lesion symptom mapping to a large cohort of patients with histologically confirmed intracranial meningiomas. By combining spatial localization with detailed histological subtyping, we aim to delineate the anatomical distribution of meningioma subtypes in a statistically robust, unbiased manner. By comparing the spatial profile of different subtypes to known radiogenomic mutations maps, we intend to explore the spatial-genomic-histologic landscape of meningiomas and provide novel insights into their pathophysiology, with potential implications for classification, prognosis, and targeted therapeutic strategies.

## Materials and methods

### Patient population

This retrospective observational single center study enrolled 1175 patients who underwent surgical treatment of intracranial meningioma between 2007 and 2017 at our institution. 737/1175 (63%) of cases met the inclusion criteria defined as sufficient quality of preoperative magnetic resonance imaging (MRI), first-line treatment and complete clinical information about histopathological results. Eleven patients (1.5%) with intraventricular meningiomas were excluded. The clinical data was acquired from medical records including age at diagnosis, gender and histopathological findings. Tumor locations were evaluated based on preoperative MRI by experienced, board-certified skull base neurosurgeons. Histopathological diagnosis was reclassified according to the WHO 2021 classification.^17^ Details of the clinical and demographic characteristics are depicted in Table 1. This study was approved by the local ethics committee of the Medical Faculty of the Eberhard Karls University Tuebingen (No. 702/2024B02) and performed in accordance with the Declaration of Helsinki. All participants gave written informed consent. The results are reported following the STROBE guidelines.^18^

**Table 1:** Clinical and spatial characteristics of the patient cohort

	*N* = 737
**Age (years)**	57.0 ± 12.7
**Sex**	
Female	540 (73)
Male	197 (27)
**Volume (mL)**	34.2 ± 40.3
**Location**	
**Non-skull base**	302 (41)
Parasagittal	115 (16)
Falcine	47 (6)
Cerebral convexity	140 (19)
**Skull base**	435(59)
Cavernous sinus	14 (2)
Sphenoid wing ^a^	58 (8)
Clinoidal	62 (8)
Tuberculum sellae	53 (7)
Planum sphenoidale	14 (2)
Olfactory groove	33 (5)
Petroclival	48 (7)
CPA	58 (8)
Cerebellar convexity	16 (2)
Tentorial	28 (4)
Foramen jugulare	11 (2)
Foramen magnum	13 (2)
Temporobasal	10 (1)
Frontobasal	16 (2)
Histology	
Meningothelial	372 (51)
Fibrous	58 (8)
Transitional	85 (12)
Psammomatous	20 (3)
Angiomatous	18 (2)
Microcytic	19 (3)
Secretory	29 (4)
lymphoplasmacyte	0 (0)
Metaplastic	6 (1)
Chordoid	12 (2)
Clear cell	1 (0)
Papillary	0 (0)
Rhabdoid	2 (0)
Atypical	113 (15)
Anaplastic	2 (0)
WHO	
CNS Grade 1	579 (79)
CNS Grade 2	156 (21)
CNS Grade 3	2 (0)

CNS, central nervous system; CPA, cerebellopontine angle; WHO, World Health Organization.

This table summarizes key demographic and anatomical data for the present patient cohort. The percentage of the total cohort is shown in parentheses.

^a^Medial/lateral sphenoid wing.

### Magnetic resonance imaging

Imaging studies were conducted at our institution using 1.5 T MRI scanner (MRI; Siemens Healthineers) including a high-resolution T1-weighted contrast-enhanced MPRAGE sequence (slice thickness: 1.0 mm; TR: 2300 ms; TE: 3.51 ms; TI: 1100; flip angle: 8°; pixel bandwidth: 130; pixel spacing: 1/1 mm; matrix: 256 × 256). Nevertheless, many of the segmentations were carried out on preoperative MRI that have been performed externally, therefore the above listed parameters may vary significantly among patients. The last scan before surgery was used for analyses.

### Voxel-based lesion symptom mapping (VLSM)

**Figure 1:**
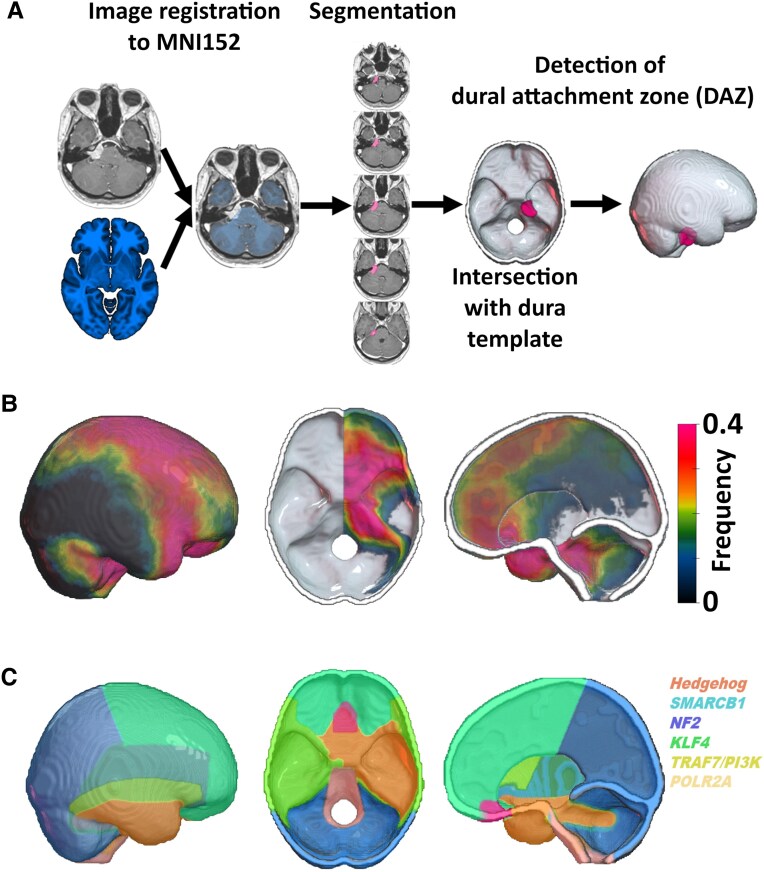
**Workflow.** (**A**) Patients’ individual MRI data were registered to a 1-mm isotropic, high-resolution, T1-weighted MRI template provided by the Montreal Neurological Institute (MNI). Meningiomas were semi-automatically segmented and flipped to the right hemisphere. Tumor masks were intersected with a standardized template representing the dura and falx to detect the dural attachment zone (DAZ) of the meningioma. This information was used for the voxel-wise lesion symptom mapping analysis. (**B**) Frequency maps represent the spatial distribution of meningioma DAZ in the present cohort (*n* = 737). Warmer colors indicate higher tumor frequency across the cohort, highlighting anatomical hotspots. Meningiomas were most frequently located at the anterior two-thirds of the falx cerebri, the frontal convexity, the sphenoid wing, and the petroclival junction. In contrast, tumors were rarely observed in the parieto-occipital and cerebellar convexity. This map provides a reference for the overall anatomical distribution of meningiomas prior to subtype-specific stratification. (**C**) Schematic radiogenomic mutation maps illustrating the known anatomical predilection of meningiomas harboring common driver mutations. These binary maps were manually created based on anatomical descriptions in the literature^3,7,9,10,12,13,24-31^ and used for voxel-wise spatial concordance analysis with histological subtypes.

All Digital Imaging and Communications in Medicine (DICOM) format images were first converted to the Neuroimaging Informatics Technology Initiative (NIfTI) format by using *dcm2niix.*^19^ Statistical Parametric Mapping Software version 12 (SPM12, Institute of Neurology, University College London, London, UK; https://www.fil.ion.ucl.ac.uk/spm/docs/) and MATLAB (R2024a, MathWorks, Natick, MA, USA) were used to register and normalize patient’s MR images to a standard brain template (MNI152; Montreal Neurological Institute, McGill University, Montreal, Quebec, Canada) (Fig. 1). The registration was visually assessed, and a manual correction was applied if required. The normalized image was resampled to a voxel size of 1 × 1 × 1 mm. The tumor was semi-automatically segmented using a fast-marching method implemented in MATLAB (*see* Supplementary material). The segmentation was performed and reviewed by two neurosurgeons. Manual corrections to the tumor mask were made in MRIcron (https://www.nitrc.org/projects/mricron/). The individual tumor volume was noted, and the tumor mask was saved for further analysis. All tumor masks were mirrored to the right hemisphere of the brain. Dural attachment zone (*DAZ*) was acquired by intersecting the tumor mask with a template representing the dura, falx and tentorium. This template was generated based on a publicly available high-resolution computer tomography (CT) template^20^ and falx/tentorium masks provided by *Brain Biomechanics Imaging Resources Project* (https://www.nitrc.org/projects/bbir). Voxel-based lesion symptom mapping (VLSM) enables to evaluate the relationship of a predictor (e.g. DAZ of the meningioma) to a specific outcome or feature (e.g. histopathological subtype) at individual voxels (i.e. voxel-vice).^21^ In this study, VLSM was based on a univariable linear regression model and one-tail t-statistics as implemented in SPM12 (https://www.fil.ion.ucl.ac.uk/spm) and NiiStat (https://www.nitrc.org/projects/niistat). Categorial variables were incorporated after dummy coding (0: reference group; 1: observational group). Only voxels affected in at least 10 patients were included in the VLSM analysis to ensure sufficient statistical power and to minimize the influence of outliers.^22^ Thus, 193 228 voxels were included in the VLSM analysis. VLSM statistics provides *Z*-scores representing standardized test statistics derived from these models. A positive *Z*-score indicates a positive association between the occurrence of meningiomas in this voxel and a specific feature (e.g. a specific histopathological subtype). To account for multiple comparisons across the numerous voxels analyzed, False Discovery Rate (FDR) correction is applied. This statistical method controls the expected proportion of false positives among the voxels identified as significant, enhancing the reliability of the results.^22,23^

### Predictive modeling of histology and grade

To determine whether tumor localization carries predictive value for histological subtype or CNS WHO grade, a multinomial logistic regression model was implemented in MATLAB (R2024a, MathWorks, Natick, MA, USA). For each patient, the individual tumor mask was intersected with the corresponding subtype-specific VLSM probability maps (*Z*-scores). The mean *Z*-value within the intersection was extracted as a spatial covariate, reflecting the degree of spatial similarity between the individual tumor and each subtype-specific spatial pattern. A regularized multinomial logistic regression model was then trained using a ridge-penalized linear learner within a one-versus-all coding framework (*see* Supplementary material). Model performance was evaluated using fivefold cross-validation to prevent overfitting and to estimate generalizability. Separate models were constructed for histological subtype classification (including subtypes represented by ≥10 patients) and CNS WHO grade prediction (Grade 1 versus Grade 2). Predictive performance was quantified by overall accuracy (ACC), per-class accuracy, and macro-averaged area under the receiver operating characteristic curve (AUC).

### Radiogenomic maps of meningioma associated mutations

Mutation-specific spatial distribution maps were generated based on previously published data describing the anatomical predilection of genetically defined meningioma subgroups.^3,7,9,12,13,24-36^ For this purpose, schematic mutation maps were constructed for the most common driver mutations in meningioma tumorigenesis, including *NF2*, *SMARCB1*, Hedgehog (*SMO*, *SUFU*) *AKT1*, *TRAF7*, *KLF4*, and *POLR2A*. Each map was manually delineated using a high-resolution MNI152 brain template, reflecting the region’s most frequently associated with each mutation (see Supplementary Table 1, Fig. 1). To quantify the spatial concordance between histological subtype-specific maps and radiogenomic mutation templates, we performed an area under the curve–receiver operating characteristic (AUC-ROC) analysis (*perfcurve.m* MATLAB function). This approach evaluates the ability of a continuous probability map (derived from VLSM) to distinguish between voxels belonging to a given mutation-specific anatomical distribution (binary mask) and those that do not. The AUC value ranges from 0.5 (no discrimination) to 1.0 (perfect discrimination), with higher values indicating greater spatial overlap. An AUC > 0.7 was considered indicative of significant spatial concordance. This voxel-wise analysis provides a robust, threshold-independent measure of the spatial correspondence between histological and radiogenomic patterns.

### Statistics

Statistical evaluation of clinical data was performed using SPSS (IBM SPSS Statistics for Windows, Version 25.0, Armonk, NY: IBM Corporation) and custom-written MATLAB scripts including MATLAB statistics toolbox. For comparison of categorical data, we used a chi-squared test (X² test). Group comparisons of metric variables were based on non-parametric Mann-Whitney U-tests. *P*-values < 0.05 were considered significant. Results are shown as mean ± standard deviation (SD).

## Supplementary Material

fcag025_Supplementary_Data

## Figures and Tables

**Table 2:** Clinical and spatial characteristics of histological meningioma subtypes

	MNG *N* = 372	FI *N* = 58	TR *N* = 85	PS *N* = 20	AN *N* = 18	MI *N* = 19	SE *N* = 29	ME *N* = 6	CH *N* = 12	AT *N* = 113
AGE	57 ± 12	54 ± 14	56 ± 13	60 ± 15	56 ± 10	52 ± 10	58 ± 11	55 ± 11	53 ± 18	**59** **±** **14**
(years)										
Female SEX	284 (76)	46 (79)	66 (78)	18 (90)	12 (67)	13 (68)	24 (83)	5 (83)	7 (58)	**60** (**53)**
male SEX	88 (24)	12 (21)	19 (22)	2 (10)	6 (33)	6 (32)	5 (17)	1 (17)	5 (42)	**53** (**47)**
CNS WHO 1	**351** (**94)**	**56** (**97)**	**82** (**96)**	**20** (**100)**	**18** (**100)**	**19** (**100)**	26 (90)	5 (83)	**0** (**0)**	**0** (**0)**
CNS WHO 2	**21** (**6)**	**2** (**3)**	**3** (**4)**	**0** (**0)**	**0** (**0)**	**0** (**0)**	3 (10)	1 (17)	**12** (**100)**	**113** (**100)**
Volume (mL)	**27** **±** **34**	41 ± 59	31 ± 32	17 ± 14	30 ± 29	38 ± 39	**15** **±** **13**	50 ± 82	**85** **±** **72**	**57** **±** **44**
Non-skull base	**99** (**27)**	30 (52)	42 (49)	8 (40)	8 (44)	**13** (**68)**	**5** (**17)**	3 (50)	4 (33)	**87** (**77)**
Parasagittal	**32** (**9)**	11 (19)	19 (22)	5 (25)	3 (17)	**7** (**37)**	1 (3)	1 (17)	0 (0)	**35** (**31)**
Falcine	18 (5)	**8** (**14)**	9 (11)	2 (10)	0 (0)	1 (5)	0 (0)	0 (0)	0 (0)	8 (7)
Cerebral Convexity	**49** (**13)**	11 (19)	14 (16)	1 (5)	5 (28)	5 (25)	4 (14)	2 (33)	4 (33)	**44** (**39)**
Skull base	**273**(**73)**	28 (48)	43 (51)	12 (60)	10 (66)	**6** (**32)**	**22** (**76)**	3 (50)	8 (67)	**26** (**23)**
Cavernous sinus	8 (2)	1 (2)	0 (0)	2 (10)	0 (0)	0 (0)	2 (7)	0 (0)	0 (0)	1 (1)
Sphenoid wing*	**38** (**10)**	**0** (**0)**	4 (5)	0 (0)	0 (0)	1 (5)	**6** (**21)**	0 (0)	3 (25)	5 (4)
Clinoidal	**45** (**12)**	**0** (**0)**	5 (7)	0 (0)	3 (17)	0 (0)	4 (14)	1 (17)	0 (0)	**3** (**3)**
Tuberculum sellae	**48** (**13)**	**0** (**0)**	1 (1)	1 (5)	0 (0)	1 (5)	1 (3)	0 (0)	0 (0)	**1** (**1)**
Planum sphen.	**11** (**3)**	0 (0)	1 (1)	0 (0)	1 (6)	0 (0)	0 (0)	0 (0)	0 (0)	1 (1)
Olfactory groove	**23** (**6)**	0 (0)	3 (4)	0 (0)	0 (0)	0 (0)	0 (0)	1 (17)	**3** (**25)**	3 (3)
Petroclival	**35** (**9)**	**0** (**0)**	3 (4)	2 (10)	0 (0)	1 (5)	3 (10)	0 (0)	0 (0)	**3** (**3)**
CPA	**21** (**6)**	**13** (**22)**	**12** (**14)**	**5** (**25)**	1 (6)	1 (5)	5 (17)	0 (0)	0 (0)	**0** (**0)**
Cerebellar convex.	**1** (**0)**	**6** (**10)**	**5** (**6)**	0 (0)	0 (0)	0 (0)	0 (0)	0 (0)	0 (0)	4 (4)
Tentorial	12 (3)	**6** (**10)**	5 (6)	0 (0)	0 (0)	1 (5)	1 (3)	0 (0)	1 (8)	2 (2)
Foramen jugulare	8 (2)	1 (2)	0 (0)	0 (0)	**2** (**11)**	0 (0)	0 (0)	0 (0)	0 (0)	0 (0)
Foramen magnum	8 (2)	0 (0)	1 (1)	**2** (**10)**	1 (6)	0 (0)	1 (3)	0 (0)	0 (0)	0 (0)
Temporobasal	4 (1)	1 (2)	2 (2)	0 (0)	1 (6)	1 (5)	0 (0)	0 (0)	0 (0)	1 (1)
Frontobasal	11 (3)	0 (0)	1 (1)	0 (0)	1 (6)	0 (0)	1 (3)	0 (0)	1 (8)	1 (1)

AN, angiomatous; AT, atypical; CH, chordoid; FI, fibrous; ME, metaplastic; MI, microcytic; MNG, meningothelial; PS, psammomatous; SE, secretory; TR, transitional.

This table summarizes key demographic and anatomical data for each histological meningioma subtype in the cohort. Percentages (shown in parentheses) refer to the proportion of patients within each histological subgroup. Each subgroup data was compared statistically to the remaining cohort (Mann-Whitney U and Chi-square tests). Significant differences (*P* < 0.05) are indicated in bold.

**Figure 2:**
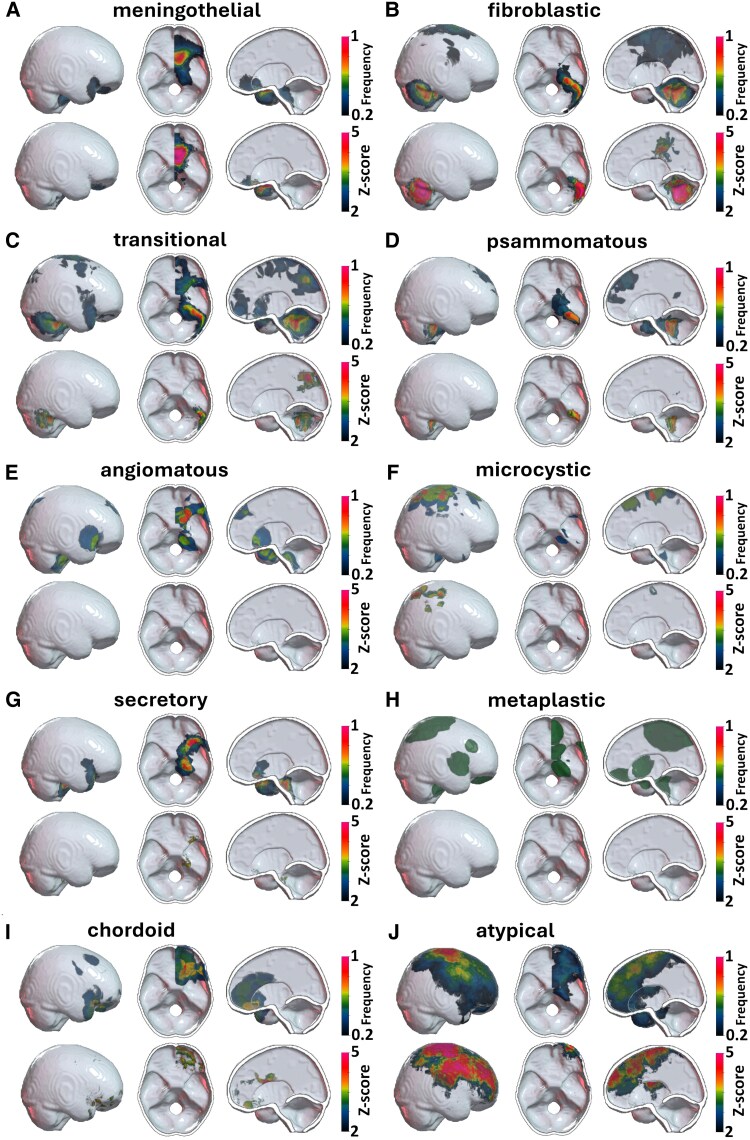
**Voxel-based spatial distribution of histological meningioma subtypes.** The figure shows the results of VLSM for the most common histological subtypes of meningioma, projected onto the Montreal Neurological Institute (MNI152) template. Each map illustrates the frequency map (first row) and the statistically association as assessed by voxel-based linear regression (second row) to a specific meningioma subtype (i.e. *Z*-values). Significant *Z*-scores are thresholded and corrected for multiple comparisons (*P* < 0.05; FDR corrected). Distinct spatial patterns were observed for (**A**) meningothelial (*N* = 372), (**B**) fibrous (*N* = 58), (**C**) transitional (*N* = 85), (**D**) psammomatous (*N* = 20), (**F**) microcystic (*N* = 19), (**G**) secretory (*N* = 29), (**I**) chordoid (*N* = 12), and (**J**) atypical (*N* = 113) meningiomas. However, there were no significant clusters for (**E**) angiomatous (*N* = 18), (**H**) metaplastic (*N* = 6) subtypes.

**Figure 3:**
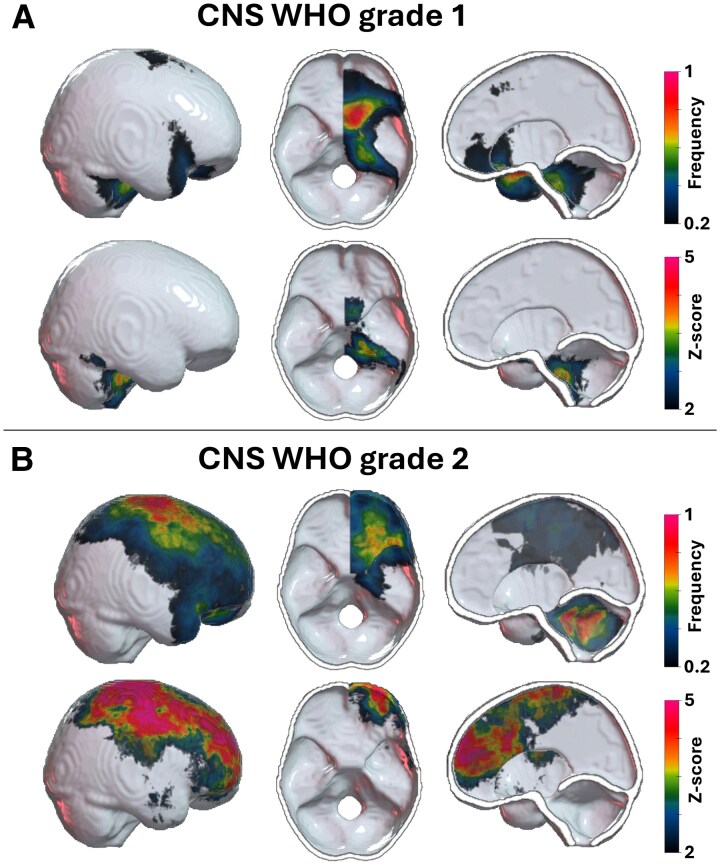
**Voxel-based spatial distribution of CNS WHO Grade 1 and Grade 2 meningiomas.** The figure illustrates the results of VLSM comparing the anatomical predilection of CNS WHO Grade 1 (*N* = 579) and Grade 2 (*N* = 156) meningiomas. Tumor masks were registered to the Montreal Neurological Institute (MNI152) template space, and statistical mapping identified regions significantly associated with each grade. Each panel illustrates the frequency map (first row) and the statistically association as assessed by voxel-based linear regression (second row) to a specific meningioma subtype (i.e. *Z*-values). Significant *Z*-scores are thresholded and corrected for multiple comparisons (*P* < 0.05; FDR corrected). (**A**) CNS WHO Grade 1 meningiomas were predominantly located at the anterior and central skull base, including the planum sphenoidale, tuberculum sellae, sphenoid wing, clinoid process, petrous bone, and clivus. (**B**) In contrast, CNS WHO Grade 2 meningiomas showed a significant preference for non-skull base locations, particularly the frontal convexity, frontobasal region, and anterior two-thirds of the falx cerebri.

**Table 3:** Multiclass performance of the histological subtype prediction model

Histological subtype	Precision	Recall	F1
Meningothelial	0.6075	0.8280	0.7008
Fibrous	0.3846	0.2542	0.3061
Transitional	0.4681	0.2529	0.3284
Psammomatous	0.3000	0.1500	0.2000
Angiomatous	0.3333	0.1111	0.1667
Microcystic	0.4167	0.2632	0.3226
Secretory	0.0000	0.0000	0.0000
Chordoid	0.4000	0.1667	0.2353
Atypical	0.3846	0.3448	0.3636

Overall metrics: Accuracy = 0.5423, Macro-AUC = 0.7461, Macro-F1 = 0.2915, Micro-F1 = 0.5423, Balanced Accuracy = 0.2634, Cohen’s κ = 0.2526 (5-fold cross-validation).

**Table 4:** Binary performance of CNS WHO grade prediction model

CNS WHO grade	Precision	Recall	F1
Grade 1	0.8030	0.9373	0.8650
Grade 2	0.4194	0.1646	0.2364

Overall metrics: Accuracy = 0.7705, Macro-AUC = 0.7542, Macro-F1 = 0.5507, Micro-F1 = 0.7705, Balanced Accuracy = 0.5509, Cohen’s κ = 0.1306 (5-fold cross-validation).

**Table 5:** Voxel-based spatial concordance (AUC-ROC) between histological meningioma subtypes, WHO grades, and known mutation-specific radiogenomic maps from literature

Mutation subtype	Hedgehog SMO/SUFU	NF2	SMARCB1	KLF4	TRAF7/PI3K	POLR2A
Meningothelial	**0**.**96**	0.29	0.42	0.65	**0**.**82**	**0**.**90**
Fibrous	0.11	**0**.**83**	0.39	0.44	0.30	0.17
Transitional	0.19	**0**.**87**	0.28	0.46	0.38	0.44
Psammomatous	0.04	0.60	0.47	0.46	0.51	**0**.**80**
Angiomatous	0.38	0.57	0.40	0.50	0.51	0.63
Microcystic	0.07	0.63	0.51	0.42	0.31	0.39
Secretory	0.12	0.49	0.25	**0**.**75**	**0**.**80**	**0**.**88**
Metaplastic	0.64	0.68	0.36	0.41	0.38	0.57
Chordoid	**0**.**92**	0.34	0.64	0.48	0.47	0.18
Atypical	0.22	0.35	**0**.**86**	0.35	0.12	0.09
WHO Grade 1	0.63	0.67	0.10	**0**.**71**	**0**.**87**	**0**.**94**
WHO Grade 2	0.38	0.33	**0**.**90**	0.29	0.13	0.06

The table displays area under the curve (AUC) values quantifying voxel-wise spatial overlap between subtype-specific probability maps and schematic binary mutation maps for the most common driver mutations in meningiomas (Hedgehog, NF2, SMARCB1, KLF4, TRAF7/PIK3, POLR2A). AUC values >0.7 are considered indicative of significant spatial concordance and are highlighted in bold. The table also includes spatial concordance values for WHO Grade 1 and Grade 2 meningiomas.

## Data Availability

The data that support the findings of this study are available from the corresponding author upon reasonable request. All custom MATLAB scripts used for tumor segmentation as well as the scripts used for the predictive modeling and AUC–ROC analysis, are provided in the Supplementary material. For VLSM, no external or proprietary code was used beyond standard SPM12 and NiiStat functions.

## Results

### Patient cohort and tumor characteristics

The present retrospective study enrolled 737 consecutive patients (age: 57.0 ± 12.7 years) undergoing primary surgical treatment of an intracranial meningioma. Overall, there was a predominance of female sex (540/737, 73%). Mean tumor volume was 34.2 ± 40.3 mL. 435/737 (59%) of meningiomas were located at the skull base. Meningothelial meningiomas were the most common histological subtype (372/737, 51%). Lymphoplasmacyte-rich (*n* = 0), clear cell (*n* = 1), papillary (*n* = 0), rhabdoid (*n* = 2) and anaplastic (*n* = 2) subtypes were too rare for further statistical evaluation. 579/737 (79%) of cases were assigned to a CNS WHO Grade 1. Full details are summarized in Table 1. Comparing the histological subtypes, there were no major group differences in patient age although patients with atypical meningiomas were significantly older than the remaining cohort (*H* = 2.29, *P* = 0.022). Predominance of female sex was observed in all histological subtypes. However, atypical (53/113, 47% male) subtypes were more frequent in male patients compared with the remaining cohort (144/624, 23%; X² = 27.73, *P* < 0.001). None of the histological subtypes exhibited an exclusive preference for either skull base or non-skull base location. However, meningothelial and secretory meningiomas were more frequent in skull-base locations compared with the remaining cohort. In contrast, microcytic and atypic meningiomas were predominantly found in non-skull base locations (i.e. parasagittal and cerebral convexity). Detailed characteristics of the histological subtypes are summarized in Table 2. In general, CNS WHO Grade 2 meningiomas presented less sex disparity (22% versus 46% male; X² = 38.12, *P* < 0.001), larger tumor volumes (57.3 ± 48.2 and 34.2 ± 35.5 ml; H = 8.76, *P* < 0.001) and a preference for non-skull base locations (70% versus 33%; X² = 65.31, *P* < 0.001) compared with CNS WHO Grade 1 meningiomas.

### Spatial distribution of meningioma subtypes

After each individual preoperative MR image was registered to the MNI152 template space, meningiomas were segmented manually. Tumor masks were aggregated to calculate the spatial distribution of all included meningiomas. The resulting frequency maps revealed meningioma hotspots in the anterior two-third of the falx cerebri, the frontal convexity including the frontoparietal junction, and skull base structures such as the sphenoid wing and petroclival junction. In contrast, only a few lesions were observed in the parieto-occipital convexities (Fig. 1).

To assess subtype-specific spatial preferences, voxel-based lesion symptom mapping (VLSM) was performed to identify statistically significant clusters. Meningothelial meningiomas demonstrated significant clustering along the midline of the anterior and middle skull base, including the planum sphenoidale, tuberculum sellae, greater and lesser sphenoid wings, clinoidal process, and clivus. Fibrous meningiomas were predominantly located in the region of the petrosal bone, the adjacent squamous part of the occipital bone, the tentorium, and the posterior third of the falx cerebri. Transitional meningiomas shared spatial features with both meningothelial and fibrous subtypes, with significant clustering in the petrous bone, occipital squama, tentorium, and posterior falx. Psammomatous meningiomas occurred across a broader anatomical range, including the petrous bone, clivus, tuberculum sellae, foramen magnum, and both the anterior and posterior segments of the falx. VLSM identified significant clusters in the petrous bone and the posterior third of the falx. Angiomatous meningiomas exhibited a spatial pattern similar to that of the meningothelial subtype; however, VLSM did not reveal any statistically significant clustering. Secretory meningiomas showed distinct spatial clustering at the lateral aspects of the greater and lesser sphenoid wings and the petroclival junction. Microcystic meningiomas were predominantly localized around the parasagittal cerebral convexity, particularly at the frontoparietal junction. Metaplastic meningiomas did not show any consistent spatial clustering, likely due to the small sample size in the present cohort (*n* = 6). Chordoid meningiomas clustered significantly in the frontobasal region and the anterior third of the falx. Atypical meningiomas demonstrated a clear preference for the frontal convexity and the anterior two-thirds of the falx cerebri. The VLSM results for all histological subtypes are summarized in Fig. 2.

### Spatial distribution of CNS WHO grades

We also applied VLSM to determine the spatial pattern of CNS WHO Grade 1 and Grade 2 meningiomas (Fig. 3). Skull base meningiomas covering the planum sphenoidale, tuberculum sellae, greater and lesser sphenoid wings, clinoidal process, clivus and the petrous bone were significantly more likely to represent CNS WHO Grade 1 meningiomas. In contrast, meningiomas of the lateral frontobasal region, the frontal convexity and the anterior two-thirds of the falx cerebri were associated with CNS WHO Grade 2 meningioma.

### Predictive modeling of histology and grade

To explore whether tumor localization can predict histopathological characteristics, we performed a multinomial logistic regression using the mean voxel-wise *Z*-value obtained from the intersection of each individual tumor mask with subtype-specific distribution maps. This model achieved overall accuracy = 0.542, macro-AUC = 0.746, macro-F1 = 0.292, micro-F1 = 0.542, balanced accuracy = 0.263, and Cohen’s κ = 0.253 (5-fold cross-validation). Per-class performance varied: meningothelial tumors were identified with high recall (0.828, F1 = 0.701), while other histological subtypes showed modest F1 scores (Table 3). Overall, location alone provides limited discriminatory power for histological subtype beyond the dominant meningothelial class, consistent with class imbalance and biological overlap among non-meningothelial phenotypes. When applying the same analysis to predict CNS WHO grading, this binary model achieved an better performance with an accuracy = 0.771, macro-AUC = 0.754, macro-F1 = 0.551, micro-F1 = 0.771, balanced accuracy = 0.551, and Cohen’s κ = 0.131 (5-fold cross-validation). Per-class performance showed CNS WHO Grade 1 was identified with high recall (0.937, F1 = 0.865) while CNS WHO Grade 2 exhibited lower recall (0.165, F1 = 0.236), consistent with class imbalance and partial spatial overlap between grades. Overall, spatial localization alone provides useful discriminative signal for grading, particularly for CNS WHO Grade 1 (Table 4).

### Relationship between spatial pattern of meningioma subtypes and radiogenomic maps

To quantify spatial concordance, we performed an AUC-ROC analysis, comparing the voxel-wise probability maps of histological meningioma subtypes with binary topographic mutation maps, described in literature.^3,7,9,10,12,13,24-31^ The AUC value served as a measure of how well the spatial distribution of a given histological subtype predicted the presence of a specific mutation across standardized brain space. AUC values >0.7 were considered indicative of significant spatial concordance (Table 5). The topography of Hedgehog (SMO/SUFU) mutations (i.e. medial anterior skull base) correlated with the occurrence of meningothelial and chordoid subtypes. The NF2 mutation map (i.e. parieto-occipital cerebral convexity, falx and tentorium, cerebellar convexity) showed a spatial concordance with the fibrous and transitional subtypes. The spatial signature of SMARB1 associated meningiomas (i.e. frontal cerebral convexity, lateral anterior skull base, anterior two-thirds of the falx) was linked to aggressive atypical subtypes. KLF4 mutation (i.e. medial and lateral middle skull base) and TRAF7 maps (i.e. medial skull base) were associated to secretory meningiomas. TRAF7 was also related to the meningothelial subtype. The POLR2A mutation maps (i.e. medial posterior skull base) showed a spatial concordance to meningothelial, psammomatous and secretory meningiomas. In general, CNS WHO Grade 1 meningiomas showed a spatial signature resembling the KLF4, TRAF7 and POLR2A mutation maps, while CNS WHO Grade 2 meningiomas showed an excellent overlap with the SMARCB1 mutation map.

## Discussion

This is the first study using voxel-based lesion mapping (VLSM) to analyze and describe the spatial distribution of intracranial meningioma histological subtypes. Although there is a good understanding of the histological grading of meningiomas, the topographic distribution of meningiomas has not been extensively studied. The present study provides evidence for a significant clustering of specific histological phenotypes which showed a remarkable correspondence to known radiogenomic maps of driver mutations in meningioma tumorigenesis.

The demographic characteristics of our cohort resembled other large-scale meningioma cohorts.^10,37-39^

Because a standardized anatomical framework for meningioma distribution is lacking, comparison of spatial patterns across these studies remains, however, difficult. The present voxel-based approach (VLSM) allows a sub-millimetric localization of tumor predilection zones across cohorts. This approach eliminates subjectivity inherent to manual region labelling and enables rater-independent, reproducible localization patterns. To date, only three studies have applied voxel-based spatial analyses to describe the anatomical distribution of intracranial meningiomas. Despite methodological differences, they consistently identify predilection sites along the midline parasagittal region, with a hotspot at the anterior two-thirds of the superior sagittal sinus and falx, the frontobasal region, and the cerebellopontine angle, while meningiomas are rarely found at the parietal convexity.^14-16^ The present study is the first to systematically evaluate and describe the spatial profile of different histological subtypes confirming and refining the available evidence.^28-31^

A key strength of our voxel-based methodology is its capacity to reveal novel spatial clustering patterns of meningiomas that have likely been overlooked by traditional descriptive or categorical localization methods. While earlier studies noted a general non-skull-base preference for higher-grade tumors,^15,16,39,40^ our voxel-wise approach revealed a previously undescribed clustering of CNS WHO Grade 2 meningiomas at the frontoparietal junction of the cerebral convexity and along the anterior two-thirds of the falx cerebri. This finding suggests that the transition between anterior and middle meningeal compartments may constitute a biologically distinct dural microenvironment favoring atypical transformation—an insight with potential implications for tumorigenesis and recurrence risk. From an embryological perspective, the frontoparietal junction (i.e. coronal suture and anterior fontanel) occupies a special position within the cerebral convexity. Here, neural-crest derived progenitor cells (NCC) of the frontal meninges undergo epithelial-to-mesenchymal transition (EMT) to contribute to cranial bone and suture formation.^41-43^ Close reciprocal interactions of NCC with parietal mesodermal cells via paracrine signal pathways (such as Wnt) are known to regulate osteogenesis within the frontoparietal junction.^43^ Interestingly, EMT-like processes are considered to enable meningioma cells to infiltrate surrounding structures,^43-46^ a feature of high-grade meningioma. This pathophysiological overlap of EMT processes in the development of the coronal suture and the aggressiveness of meningiomas might explain the predominance of CNS WHO Grade 2 meningiomas at the frontoparietal junction.

Furthermore, our voxel-based approach ultimately provides anatomically precise distribution maps which could be used for further radiogenomic studies. Numerous studies have characterized the genomic landscape of driver mutations in meningioma tumorigenesis, consistently demonstrating that specific genetic alterations are associated with distinct anatomical predilections and specific histological subtypes.^3,7,9,10,12,13,24-27^ This evident spatial predominance of driver mutations lead to the development of schematic radiogenomic ‘mutation maps’.^9^ Since then, these maps have been reproduced and refined in subsequent studies.^3,10,32-36^ Our study is the first to combine voxel-based spatial statistics with published mutation-specific radiogenomic maps, quantitatively assessing their spatial concordance. We observed a remarkable spatial concordance between histological subtypes and mutation-specific distribution maps, confirming previously described histogenomic associations. Only a few exceptions were noted, which may either reflect methodological variation or suggest potential novel associations that warrant further investigation. Both, psammomatous and secretory meningiomas correlated with the POLR2A template (i.e. Tuberculum sellae, clivus, Foramen magnum), an association not known from literature. A recent study on 62 Foramen magnum meningiomas describes a predominance of TRAF7 (∼42%) and AKT1 (∼31%) mutations. POLR2A was found in ∼13% of the cases, all displaying meningothelial histology.^47^ In our study, chordoid meningiomas showed a spatial association to the Hedgehog template—unknown for this subtype.^48-50^ In contrast, a recent study described EP400, KMT2C and KMT2D mutations in chordoid meningiomas.^48^ Considering their roles in neural-crest cell differentiation and craniofacial morphogenesis,^51,52^ it is plausible that these factors might contribute to the tumorigenesis of meningiomas of the anterior skull base.

We further evaluated the predictive value of the voxel-based distribution maps for histological subtype and CNS WHO grade. Using a prediction model based solely on the spatial overlap between each individual tumor mask and the subtype-specific voxel maps, we found a moderate performance for histological, but a markedly better accuracy for CNS WHO grade prediction. These findings indicate that anatomical localization alone contains measurable discriminative information, particularly for distinguishing CNS WHO Grade 1 from CNS WHO Grade 2 tumors. This observation supports our central hypothesis that meningioma location is not random but reflects underlying biological and molecular properties, as demonstrated by the spatial concordance between histological and radiogenomic maps. The predictive modeling thus provides quantitative evidence that the spatial component can serve as a surrogate marker for tumor aggressiveness and might contribute to future multimodal prediction frameworks combining imaging, histology, and genomics.

The available spatial maps of driver mutations in meningioma tumorigenesis are typically schematic in nature, based on aggregated clinical observations or descriptive subgrouping, and are therefore prone to subjectivity and anatomical oversimplification. As a result, the reported mutation maps often differ considerably in their spatial resolution, anatomical detail or present data in a simplified manner.^3,10,32-36^ Against this background, the mutation maps are the most important limitation of the present study. The present study compared the spatial distribution of histological subtypes to schematic mutation maps without utilizing the individual genomic characteristics. Future studies should aim to develop voxel-based mutation maps on actual genomic data. Unlike manually drawn or qualitatively interpreted diagrams, this approach minimizes interpretive bias and enables the generation of standardized mutation templates that can be integrated into future radiogenomic research. These templates hold significant potential for advancing our understanding of the spatial biology of meningiomas, improving non-invasive genotype prediction, and informing image-guided treatment planning and personalized risk stratification.

## Conclusion

This study provides the first voxel-based analysis of the spatial distribution of histological meningioma subtypes, revealing distinct anatomical predilections that align with known radiogenomic mutation patterns. By integrating predictive modeling, we further demonstrate that the spatial component itself holds diagnostic value for histopathological and grading prediction, underlining the biological significance of tumor topography. By demonstrating a high degree of spatial concordance between histological subtypes and driver mutation maps, our findings confirm established histogenomic associations and suggest previously unrecognized relationships. The standardized, stereotactically normalized spatial templates generated in this study offer an anatomically precise and bias-reduced foundation for future radiogenomic research. These maps may facilitate non-invasive prediction of tumor genotype, enhance molecular stratification, and support more personalized approaches to diagnosis and treatment planning in meningioma care.
