# Supplementary material for: Voxel-based spatial distribution of intracranial meningioma subtypes and their relationship to radiogenomic maps
Source: Brain Commun. 2026 Jan 30;8(1):fcag025. doi: 10.1093/braincomms/fcag025 (PMC12947794; doi:10.1093/braincomms/fcag025)
Supplement: fcag025_Supplementary_Data [file fcag025_supplementary_data.docx]

| **Gene** | **Location** | **Protein** | **Gene status** | **Mutation map** | **associated**  **subtype** | **CNS WHO grades** | **references** |
| --- | --- | --- | --- | --- | --- | --- | --- |
| **SMO** | 7p32.1 | Smoothened | Oncogene | Anterior medial skull base | meningothelial | 1 | ^1–6^ |
| **SUFU** | 10q24.32 | Suppressor of fused homolog | Tumor suppressor | familial multiple meningiomas | meningothelial | 1 | ^7–9^ |
| **NF2** | 22a12.2 | Merlin | Tumor  Suppressor | falx cerebri  tentorium cerebelli  cerebral convexity  cerebellar convexity | fibrous  psammomatous  transitional  atypical | 1-3 | ^10–18^ |
| **SMARCB1** | 22a11.23 | SWI/SNF-related matrix-associated actin-dependent regulator of chromatin subfamily B member 1 | Tumor suppressor | anterior third of the falx  frontal cerebral convexity | fibrous  transitional  atypical | 1-3 | ^19–22^ |
| **TRAF7** | 16p13.3 | TNF receptor-associated factors 7 | Tumor  Suppressor | anterior and middle medial skull base | meningothelial  secretory | 1 | ^1,17,23–25^ |
| **PIK3CA** | 3q26.32 | Phosphatidylinositol-4,5-bisphosphate 3-kinase, catalytic subunit alpha (p110α protein) | Oncogene | anterior and middle medial skull base | meningothelial  transitional | 1 | ^3,26–29^ |
| **KLF4** | 9p31 | Krüppel-like factor 4 | Tumor suppressor | middle and lateral skull base | secretory | 1 | ^1,17,24,30–32^ |
| **POLR2A** | 17p13.1 | RNA polymerase II subunit A | Oncogene | tuberculum sellae  middle and posterior medial skull base | meningothelial | 1 | ^1,17,33,34^ |
| **AKT1** | 14q32.33 | Protein kinase B alpha, beta, and gamma | Oncogene | anterior and middle medial skull base | meningothelial  transitional | 1 | ^2–4,17,35^ |

**Supplementary Table 1:** Genes implicated in meningiomas and meningioma development

**References**

1. Clark VE, Erson-Omay EZ, Serin A, et al. Genomic analysis of non-NF2 meningiomas reveals mutations in TRAF7, KLF4, AKT1, and SMO. *Science (1979)*. 2013;339(6123):1077-1080. doi:10.1126/SCIENCE.1233009

2. Brastianos PK, Horowitz PM, Santagata S, et al. Genomic sequencing of meningiomas identifies oncogenic SMO and AKT1 mutations. *Nat Genet*. 2013;45(3):285-289. doi:10.1038/NG.2526

3. Abedalthagafi M, Bi WL, Aizer AA, et al. Oncogenic PI3K mutations are as common as AKT1 and SMO mutations in meningioma. *Neuro Oncol*. 2016;18(5):649-655. doi:10.1093/NEUONC/NOV316

4. Strickland MR, Gill CM, Nayyar N, et al. Targeted sequencing of SMO and AKT1 in anterior skull base meningiomas. *J Neurosurg*. 2017;127(2):438-444. doi:10.3171/2016.8.JNS161076

5. Boetto J, Bielle F, Sanson M, Peyre M, Kalamarides M. SMO mutation status defines a distinct and frequent molecular subgroup in olfactory groove meningiomas. *Neuro Oncol*. 2017;19(3):345-351. doi:10.1093/NEUONC/NOW276

6. Findakly S, Choudhury A, Daggubati V, Pekmezci M, Lang UE, Raleigh DR. Meningioma cells express primary cilia but do not transduce ciliary Hedgehog signals. *Acta Neuropathol Commun*. 2020;8(1). doi:10.1186/S40478-020-00994-7

7. Aavikko M, Li SP, Saarinen S, et al. Loss of SUFU function in familial multiple meningioma. *Am J Hum Genet*. 2012;91(3):520-526. doi:10.1016/J.AJHG.2012.07.015

8. Pathmanaban ON, Sadler K V., Kamaly-Asl ID, et al. Association of genetic predisposition with solitary schwannoma or meningioma in children and young adults. *JAMA Neurol*. 2017;74(9):1123-1129. doi:10.1001/JAMANEUROL.2017.1406

9. Smith MJ, Beetz C, Williams SG, et al. Germline mutations in SUFU cause Gorlin syndrome-associated childhood medulloblastoma and redefine the risk associated with PTCH1 mutations. *Journal of Clinical Oncology*. 2015;32(36):4155-4161. doi:10.1200/JCO.2014.58.2569

10. Bachir S, Shah S, Shapiro S, et al. Neurofibromatosis type 2 (NF2) and the implications for vestibular schwannoma and meningioma pathogenesis. *Int J Mol Sci*. 2021;22(2):1-12. doi:10.3390/IJMS22020690

11. Choudhury A, Magill ST, Eaton CD, et al. Meningioma DNA methylation groups identify biological drivers and therapeutic vulnerabilities. *Nat Genet*. 2022;54(5):649-659. doi:10.1038/S41588-022-01061-8

12. Nassiri F, Liu J, Patil V, et al. A clinically applicable integrative molecular classification of meningiomas. *Nature*. 2021;597(7874):119-125. doi:10.1038/S41586-021-03850-3

13. Bi WL, Abedalthagafi M, Horowitz P, et al. Genomic landscape of intracranial meningiomas. *J Neurosurg*. 2016;125(3):525-535. doi:10.3171/2015.6.JNS15591

14. Driver J, Hoffman SE, Tavakol S, et al. A molecularly integrated grade for meningioma. *Neuro Oncol*. 2022;24(5):796-808. doi:10.1093/NEUONC/NOAB213

15. Sahm F, Schrimpf D, Stichel D, et al. DNA methylation-based classification and grading system for meningioma: a multicentre, retrospective analysis. *Lancet Oncol*. 2017;18(5):682-694. doi:10.1016/S1470-2045(17)30155-9

16. Gupte TP, Li C, Jin L, et al. Clinical and genomic factors associated with seizures in meningiomas. *J Neurosurg*. 2020;135(3):835-844. doi:10.3171/2020.7.JNS201042

17. Youngblood MW, Miyagishima DF, Jin L, et al. Associations of meningioma molecular subgroup and tumor recurrence. *Neuro Oncol*. 2021;23(5):783-794. doi:10.1093/NEUONC/NOAA226

18. Kros J, De Greve K, Van Tilborg A, et al. NF2 status of meningiomas is associated with tumour localization and histology. *J Pathol*. 2001;194(3):367-372. doi:10.1002/PATH.909

19. Gill CM, Loewenstern J, Rutland JW, et al. SWI/SNF chromatin remodeling complex alterations in meningioma. *J Cancer Res Clin Oncol*. 2021;147(11):3431-3440. doi:10.1007/S00432-021-03586-7

20. Hadfield KD, Smith MJ, Trump D, Newman WG, Evans DG. SMARCB1 mutations are not a common cause of multiple meningiomas. *J Med Genet*. 2010;47(8):567-568. doi:10.1136/jmg.2009.075721

21. Smith MJ, O’Sullivan J, Bhaskar SS, et al. Loss-of-function mutations in SMARCE1 cause an inherited disorder of multiple spinal meningiomas. *Nat Genet*. 2013;45(3):295-298. doi:10.1038/NG.2552

22. Van Den Munckhof P, Christiaans I, Kenter SB, Baas F, Hulsebos TJM. Germline SMARCB1 mutation predisposes to multiple meningiomas and schwannomas with preferential location of cranial meningiomas at the falx cerebri. *Neurogenetics*. 2012;13(1):1-7. doi:10.1007/S10048-011-0300-Y

23. Zotti T, Scudiero I, Vito P, Stilo R. The Emerging Role of TRAF7 in Tumor Development. *J Cell Physiol*. 2017;232(6):1233-1238. doi:10.1002/JCP.25676

24. Reuss DE, Piro RM, Jones DTW, et al. Secretory meningiomas are defined by combined KLF4 K409Q and TRAF7 mutations. *Acta Neuropathol*. 2013;125(3):351-358. doi:10.1007/S00401-013-1093-X

25. Preusser M, Brastianos PK, Mawrin C. Advances in meningioma genetics: novel therapeutic opportunities. *Nat Rev Neurol*. 2018;14(2):106-115. doi:10.1038/NRNEUROL.2017.168

26. Bujko M, Kober P, Tysarowski A, et al. EGFR, PIK3CA, KRAS and BRAF mutations in meningiomas. *Oncol Lett*. 2014;7(6):2019-2022. doi:10.3892/OL.2014.2042

27. Zadeh G, Karimi S, Aldape KD. PIK3CA mutations in meningioma. *Neuro Oncol*. 2016;18(5):603-604. doi:10.1093/NEUONC/NOW029

28. Cômes PC, Le Van T, Tran S, et al. Respective roles of Pik3ca mutations and cyproterone acetate impregnation in mouse meningioma tumorigenesis. *Cancer Gene Ther*. 2023;30(8):1114-1123. doi:10.1038/S41417-023-00621-2

29. Peyre M, Miyagishima D, Bielle F, et al. Somatic PIK3CA Mutations in Sporadic Cerebral Cavernous Malformations. *N Engl J Med*. 2021;385(11):996-1004. doi:10.1056/NEJMOA2100440

30. Di Giammartino DC, Kloetgen A, Polyzos A, et al. KLF4 is involved in the organization and regulation of pluripotency-associated three-dimensional enhancer networks. *Nat Cell Biol*. 2019;21(10):1179-1190. doi:10.1038/S41556-019-0390-6

31. Agnihotri S, Suppiah S, Tonge PD, et al. Therapeutic radiation for childhood cancer drives structural aberrations of NF2 in meningiomas. *Nat Commun*. 2017;8(1). doi:10.1038/S41467-017-00174-7

32. Berghoff AS, Hielscher T, Ricken G, et al. Prognostic impact of genetic alterations and methylation classes in meningioma. *Brain Pathol*. 2022;32(2). doi:10.1111/BPA.12970

33. Clark VE, Harmancl AS, Bai H, et al. Recurrent somatic mutations in POLR2A define a distinct subset of meningiomas. *Nat Genet*. 2016;48(10):1253-1259. doi:10.1038/NG.3651

34. Youngblood MW, Duran D, Montejo JD, et al. Correlations between genomic subgroup and clinical features in a cohort of more than 3000 meningiomas. *J Neurosurg*. 2019;133(5):1345-1354. doi:10.3171/2019.8.JNS191266

35. Sahm F, Bissel J, Koelsche C, et al. AKT1E17K mutations cluster with meningothelial and transitional meningiomas and can be detected by SFRP1 immunohistochemistry. *Acta Neuropathol*. 2013;126(5):757-762. doi:10.1007/S00401-013-1187-5

**Matlab functions for segmentation and Predictive modeling**

function [BW] = gn_segment(a,b)

% automatic tumor segmentation

% a: 3D matrix of MR volume

% b: 3D binary matrix (same size as a) with tumor seed

% BW: binary tumor segmentation mask

SE = strel('cube',4); b = imdilate(b,SE);

W = graydiffweight(a,logical(b));

BW = imsegfmm(W,logical(b),0.0006*thresh);

SE = strel('cube',5); BW = imclose(BW,SE);

BW = smooth3(BW,'box',5);

BW(BW<0.2)=0; BW(BW~=0)=1;

end

%% Predictive modeling of histology and grade

% X: n-by-p predictors

% y: n-by-1 categorical or integer class labels

t = templateLinear('Learner', 'logistic', ... % binary logistic base learner

'Regularization', 'ridge', ...

'Lambda',1e-4); % tune Lambda as needed

Mdl = fitcecoc(X, y, ...

'Learners', t, ...

'Coding', 'onevsall', ... % or 'onevsone'

'CrossVal','on', 'KFold', 5); % 5-fold CV

cvErr = kfoldLoss(Mdl); % classification error

[yhat,score,~] = kfoldPredict(Mdl); % CV predictions if needed

metrics = classificationReport(y, yhat, score);

function metrics = classificationReport(y, yhat, score)

% CLASSIFICATIONREPORT Multi-class metrics from true/predicted labels (and optional scores).

% Usage:

% metrics = classificationReport(y, yhat); % labels only

% metrics = classificationReport(y, yhat, score); % with score/probabilities (n-by-K)

%

% Inputs:

% y : n×1 true labels (numeric/char/string/categorical)

% yhat : n×1 predicted labels (same length as y)

% score : optional n×K matrix of scores or probabilities aligned to class order

%

% Outputs (struct):

% .labels : class labels (categorical categories)

% .confusionMatrix : K×K counts (rows = true, cols = predicted)

% .confusionMatrixNormalized : row-normalized confusion matrix

% .accuracy : overall accuracy

% .balancedAccuracy : mean recall across classes

% .perClassTable : table of Precision/Recall/F1 per class

% .macroF1, .microF1 : macro- and micro-averaged F1 (microF1==accuracy here)

% .kappa : Cohen's kappa

% .aucPerClass, .macroAUC : (if score provided) one-vs-rest AUCs

% .brier : (if probabilities provided) multi-class Brier score

% ---- 1) Categorical conversion & label alignment ----

y = categorical(y);

yhat = categorical(yhat);

% Use the union of classes present in y and yhat to define a fixed order

allCats = categories(categorical([y; yhat]));

y = categorical(y, allCats);

yhat = categorical(yhat, allCats);

labels = categorical(allCats);

% ---- 2) Confusion matrices ----

C = confusionmat(y, yhat, 'Order', labels);

% Manual row-normalization (each row sums to 1)

rowSum = sum(C,2);

Cnorm = C ./ max(rowSum,1e-12);

% ---- 3) Overall metrics ----

n = numel(y);

acc = mean(y == yhat);

K = numel(labels);

Tp = diag(C);

rowSum = sum(C,2); % actual per class

colSum = sum(C,1)'; % predicted per class

% Per-class precision/recall/F1

precision = Tp ./ max(colSum, eps);

recall = Tp ./ max(rowSum, eps);

F1 = 2*(precision.*recall) ./ max(precision+recall, eps);

balancedAcc = mean(recall);

macroF1 = mean(F1);

microF1 = acc; % with single-label multi-class, micro-F1 equals accuracy

% Cohen's kappa

po = acc;

pe = sum(rowSum .* colSum) / (n^2);

kappa = (po - pe) / max(1 - pe, eps);

perClassTable = table(labels, precision, recall, F1);

% ---- 4) Optional: AUCs (one-vs-rest) & Brier score if 'score' is provided ----

aucPerClass = [];

macroAUC = NaN;

brier = NaN;

if nargin >= 3 && ~isempty(score)

% Ensure numeric and correct size

if ~isnumeric(score) || size(score,1) ~= n || size(score,2) ~= K

warning('Score must be n-by-K aligned to labels. Skipping AUC/Brier.');

else

% AUCs (OVR) via perfcurve

aucPerClass = NaN(K,1);

havePerfcurve = exist('perfcurve','file') == 2;

if havePerfcurve

for i = 1:K

yi = (y == labels(i)); % logical positives

si = score(:, i); % scores for class i

try

[~,~,~,auc] = perfcurve(yi, si, true);

catch

auc = NaN;

end

aucPerClass(i) = auc;

end

macroAUC = mean(aucPerClass(~isnan(aucPerClass)));

else

warning('perfcurve not found (Statistics and Machine Learning Toolbox). Skipping AUC.');

end

% Brier score (only if scores look like probabilities)

rowSums = sum(score,2);

probsLike = all(score(:) >= -1e-12 & score(:) <= 1+1e-12) && max(abs(rowSums-1)) < 1e-6;

if probsLike

% One-hot for true labels

Yoh = zeros(n, K);

for i = 1:K

Yoh(:,i) = (y == labels(i));

end

brier = mean(sum((score - Yoh).^2, 2));

end

end

end

% ---- 5) Pack results ----

metrics = struct();

metrics.labels = labels;

metrics.confusionMatrix = C;

metrics.confusionMatrixNormalized = Cnorm;

metrics.accuracy = acc;

metrics.balancedAccuracy = balancedAcc;

metrics.perClassTable = perClassTable;

metrics.macroF1 = macroF1;

metrics.microF1 = microF1;

metrics.kappa = kappa;

metrics.aucPerClass = aucPerClass;

metrics.macroAUC = macroAUC;

metrics.brier = brier;

% ---- 6) Print a compact summary ----

fprintf('\n=== Classification Report ===\n');

fprintf('Accuracy: %.4f\n', acc);

fprintf('Balanced Acc: %.4f\n', balancedAcc);

fprintf('Macro-F1: %.4f\n', macroF1);

fprintf('Micro-F1: %.4f\n', microF1);

fprintf('Cohen''s kappa: %.4f\n', kappa);

if ~isempty(aucPerClass)

fprintf('Macro-AUC: %.4f\n', macroAUC);

end

if ~isnan(brier)

fprintf('Brier score: %.6f\n', brier);

end

fprintf('\nPer-class (rows = labels order):\n');

disp(perClassTable)

fprintf('Confusion Matrix (rows=true, cols=pred):\n');

disp(C)

fprintf('Row-normalized Confusion Matrix:\n');

disp(Cnorm)

end
